# A resource management scenario for traditional and scientific management of pink shrimp (*Farfantepenaeus paulensis*) in the Patos Lagoon estuary (RS), Brazil

**DOI:** 10.1186/1746-4269-9-6

**Published:** 2013-01-11

**Authors:** Gustavo Goulart Moreira Moura, Daniela Coswig Kalikoski, Antonio Carlos Sant’Ana Diegues

**Affiliations:** 1 Environmental Sciences at University of São Paulo (PROCAM/USP), Fishing Research Group lidership (GPP) of the Núcleo de Pesquisa e Apoio a Populações de Áreas Úmidas do Brasil (NUPAUB-USP). Rua do Anfiteatro, nº 181, Colméias - Favo 6, Cidade Universitária - CEP, 05508-060, São Paulo, SP, Brazil; 2 Natural Resources Management at University of Manitoba and Former Professor in Universidade Federal do Rio Grande (FURG), Rio Grande, Brazil; 3 Environmental Sciences at University of São Paulo (PROCAM-USP) and scientific coordinator of the Núcleo de Pesquisa e Apoio a Populações de Áreas Úmidas do Brasil (NUPAUB-USP), Sao Paulo, Brazil

**Keywords:** Traditional ecological knowledge, Traditional natural resource management systems, Modern science-based resource management, Traditional time, Incommensurability, Artisanal fishery, Patos Lagoon estuary

## Abstract

**Background:**

This article aims to discuss the incorporation of traditional time in the construction of a management scenario for pink shrimp in the Patos Lagoon estuary (RS), Brazil. To meet this objective, two procedures have been adopted; one at a conceptual level and another at a methodological level. At the conceptual level, the concept of traditional time as a form of traditional ecological knowledge (TEK) was adopted.

**Method:**

At the methodological level, we conduct a wide literature review of the scientific knowledge (SK) that guides recommendations for pink shrimp management by restricting the fishing season in the Patos Lagoon estuary; in addition, we review the ethno-scientific literature which describes traditional calendars as a management base for artisanal fishers in the Patos Lagoon estuary.

**Results:**

Results demonstrate that TEK and SK describe similar estuarine biological processes, but are incommensurable at a resource management level. On the other hand, the construction of a “management scenario” for pink shrimp is possible through the development of “criteria for hierarchies of validity” which arise from a productive dialog between SK and TEK.

**Conclusions:**

The commensurable and the incommensurable levels reveal different basis of time-space perceptions between traditional ecological knowledge and scientific knowledge. Despite incommensurability at the management level, it is possible to establish guidelines for the construction of “management scenarios” and to support a co-management process.

## Introduction

The socio-ecological approach has been widely used by several theorists of Human Ecology and Theory of commons [1] for the study and conservation of terrestrial, marine, and estuarine-lagoon ecosystems [2,3], and the cultural diversity associated with these environments [4]. Because of human occupancy and use, estuarine-lagoon systems are socio-ecological systems [2], making them an integral part of lagoon ecology and its cycles [5]. A socio-ecological co-evolution approach is needed to understand ecosystem processes, which involve the accumulation of individual and collective knowledge about the ecosystem contained in “social memories” [6]. Knowledge about ecosystems has accompanied mankind for thousands of years and through numerous environmental changes and cultural adaptations [7]. According to Toledo and Barrera-Bassols (2008), so-called traditional populations are heirs of historical experiences marked by the encounter between biological and cultural realms, and these experiences construe traditional ecological knowledge (TEK). On the other hand, modern society is dominated by its inability to remember immediate, medium-term, and long-range historical processes [4].

New studies in ethnography (or ethnoscience) have shown, since its emergence in the 1950’s, that TEK is an important source of information for the understanding of ecological processes [3,8,9]. Among those who study TEK, there are, in general, two tendencies: the followers of the complementarity/integration theory [10,11] and the followers of the “cultures meeting” theory [4,9,12-15]. The first group claims that TEK must be integrated with SK to provide insights into ecological research (biogeography, phylogeny, systematics, ethnology, population genetics, ecosystem management, etc.) [10], and have its likelihood strictly tested against reality [11]. Within this first proposal, TEK is complementary (selectively integrated) to SK, which is considered the ultimate reality against which the traditional is measured [7]. The second group proposes TEK as an alternative to Western scientific rationality [4,12], which may be linked to SK [7], and used to question [16] and transform it. In the second proposal, there is an acknowledgement of the need to change the SK paradigm, and integrate TEK, so that our “amoral, positivist society” becomes an “ethical, holistic society” ([13]: 1270).

In line with the second observed trend, a “swarm of researchers” has made a countercurrent intellectual effort to register, analyze, and reassess the TEK of traditional populations [4]. Several priority TEK fields of investigation have been identified, such as ethnotaxonomy [17], the unity and diversity of indigenous knowledge systems [5], ethno-habitats, species migration and reproduction patterns, climate, navigation and fishing skills [18], relations with the supernatural world [9], and the traditional management of natural resources [14].

Despite the great advancements in ethnosciences since the 1960’s and in research using TEK, starting from its naming and concept formation in the 1980’s [7,19], there are still several problems to be solved and questions to be answered. Among the problems noted in the scientific literature the following can be highlighted: romanticism in the use of TEK, the simplistic dichotomization between SK and TEK [20], the variability of analytical concepts used (TEK, traditional knowledge, local ecological knowledge, indigenous knowledge, etc.) [8,21] fragmentary TEK descriptions [3], and the application of academic logic to traditional populations [22]. One question unanswered question often cited in the literature, concerns the application of TEK to present “resource management scenarios” ([14]: 06, [3,8,19,21]). Some vague notes on how to apply TEK to these situations include the use of TEK as a source of biological information as well as a data source on local ecosystems [9]. Other studies acknowledge that SK and TEK are based on similar principles and are not incompatible. Also noted is the possibility for overcoming the narrowness of species-level management, commonly used in science, and the possibility of working toward an ecosystemic management approach similar to the management practiced by traditional populations [14].

This article discusses another possible and little explored solution: the incorporation of traditional time based on TEK in the construction of a local resource management scenario. Research for this case study was conducted is the Patos Lagoon estuary, located in the southernmost portion of Brazil. Despite several efforts in the study of traditional time see [23-27], these discussions fail to describe the historical see [28,29] and present importance of time control see [30] as both a tool for social control and an instrument of power. In the regulation of fishing activities, time control has been a systematic practice in management guided by conventional scientific knowledge—by prohibiting fishing during certain seasons of the year (season closures) see [9]. The restriction of time has been a systematic practice adopted by the Brazilian government in an attempt to manage fish resources by applying the so-called “closed fishing season” (see [31,32]).

The state of Rio Grande do Sul (RS), Brazil comes to the scene in the context of time control with the emergence of successive Normative Rulings, which have regulated the management of fishing resources in the Patos Lagoon estuary since the beginning 1970’s^a^. The 2004 Joint Normative Ruling (INC 2004)^b^ presently regulates fishing in the estuary. Among other regulations, INC 2004 imposes a fishing calendar for pink shrimp in the Patos Lagoon estuary. According to Kalikoski (2002), with the creation of the Forum of the Patos Lagoon (FPL), the pink shrimp fishing calendar was fashioned on the basis of scientific knowledge, and has engendered conflicts between government institutions and artisanal fisherman [33]. At the same time, selection and appropriation of fish criteria made by governmental institutions in the FPL differed from scientific recommendations. The selection and appropriation process of scientific knowledge by governmental institutions (such as the Ministry of the Environment (*Ministério do Meio Ambiente* - MMA) and Ministry of Fish and Acquaculture - Ministério da Aquicultura e Pesca e Aquacultura - MPA) guiding fishing activities is a subject for future research. In this paper, we emphasize the incorporation of traditional time in the construction of a management scenario for pink shrimp in the Patos Lagoon estuary (RS), Brazil.

The discussion of the construction of a “resources management scenario” will be done in five steps—the first two being presented in the results section and the others in the discussion section. The steps are presented as the following: 1 - Describes SK and TEK of the pink shrimp life cycle in the Patos Lagoon estuary; 2 - Describes pink shrimp management recommended by SK, and traditional pink shrimp management based on TEK, with time/calendar as an underlying analytical category; 3 - Emphasizes and defines the (in)congruencies between SK, upon which the calendar imposed by INC 2004 is based, and TEK, which is the basis for the traditional calendar/time of artisanal fishers in the estuary—both referring to the pink shrimp migration cycle in the Patos Lagoon estuary; 4 - Stresses and defines the (in)congruencies between SM and TM of pink shrimp in the Patos Lagoon estuary with time/calendar as an underlying analytical category; 5 - Based on the “cultural meeting” of different forms of knowledge, ways of management, and conceptions of time, some guidelines will be established for the construction of a possible local management scenario for pink shrimp.

In the first four sections, we will describe and discuss the observed (in)congruencies among SK and TEK and both the scientific and the traditional calendar for managing fish resources. Thus, we will test the hypothesis that knowledge and management strategies for fishing resources are incommensurable.

There are some authors, such as Menzie and Butler (2006), who call it unproductive to compare SK and TEK through the “incommensurability perspective” because it is arguably a simplistic comparison and can masquerade important “similarities” [14]. In this article, we shown that the “incommensurability” thesis is necessary to discuss at which level the (in)congruencies appear and, from here reveal what is claimed by Menzie and Butler (2006: 06) as: “The principles underlying TEK and science (that) hold similar observational principles”, which will be considered “criteria for hierarchies of validity” ([34]: 98) in the construction of a “resource management scenario” ([14]: 06). Santos *et al.* (2005) argues that no human practice could be possible if different kinds of knowledge had equal weight. Thus, from a pragmatic viewpoint, the relativism issue has a bearing on “criteria for hierarchies of validity” to solve the “epistemological problem” and make human practice possible [34]. In this case study, “criteria for hierarchies of validity” will be formulated at commensurable level(s) in order to enable the construction of “resources management scenarios;” without these criteria, such “scenarios” and management practices in such scenarios would be impossible.

Therefore, in agreement with Leff (2001), it can be understood that the incommensurability hypothesis is necessary to delimit new arrays of environmental rationality for the use of natural resources [35]. In this sense, the incommensurability hypothesis puts into action the discussion and analysis of the dialog between TEK and SK, as it does for scientific management and traditional management; and the “criteria for hierarchies of validity” provides support for the construction of a “resources management scenario”.

### Time as traditional ecological knowledge (TEK)

The basic function of any knowledge system is to organize the world [19]. Knowledge interferes in the whole process of the significance of the world and appropriation of nature [36]. From his work on popular classification systems in the 1970’s, Levi-Strauss (1976) has discussed two general ways in which scientific thinking puts the world in order: the first approximates sensitive intuition and the other is further from this intuition. The first is the practical knowledge of traditional people, or the “concrete science”, and the second is modern science [17].

In the last 50 years since the scientific panorama of the 1960’s, several definitions, naming, and thought trends have emerged in the study of the “science of the concrete”. Among the denominations used in the wider literature, the following terms can be found: “oral tradition”, “indigenous knowledge”, “local knowledge”, “community knowledge” [37], “traditional knowledge”, “traditional ecological knowledge” [21] and “ecological knowledge” [2]. To reach the goal of investigating the (in)congruencies between SK and artisanal fishers’ knowledge on the pink shrimp migration cycle in the Patos Lagoon estuary, we find the term “traditional ecological knowledge” (TEK) most adequate because: 1 - it clearly distinguishes its investigation domain, that is, nature [7,8]; 2 - it involves the perception of traditional populations about their environmental systems and how they influence and are affected by natural processes [8]; 3 - although it is not schooled by the conventional scientific paradigm, it shows its relation to ecological sciences [7,21].

A universally accepted definition of TEK does not exist in the current literature [19]. TEK can be defined in a variety of ways see [7-9,19,21]. Among them, Berkes’ (1999: 08) definition has been adopted: “… a cumulative body of knowledge, practices, and beliefs evolving through adaptative processes spread through generations by cultural transmission, about living beings’ (including humans) relations with one another and with their environments” [9]. This definition has been chosen because of its three main characteristics (practical, dynamic, and local). According to Allut (2000), the practical character of TEK provides optimum conditions for the resolution of problems that concretely emerge from the natural environment and determine cognitive needs for action. The dynamic character allows one to live in and adapt to a world which is constantly changing, and local characteristics shape interpretations of natural and social surroundings [38]—that is, its territory. These characteristics have allowed TEK to answer fundamental questions about natural resources and ecosystems by complementing, supplementing or even guiding natural sciences in the management of today’s natural resources [14].

The need to adapt to a changing world is the base of and fundamental to the construction of traditional calendars. According to Giddens (1991) and Le Goff (2003), the development of traditional calendars occurs through the observation of natural cycles (movements of the sun and moon, seasonal cycles, alternation of day and night). As such, it is possible to adopt Gardet’s (1975) perspective of time as knowledge [24,28,29].

In the modern world, time and the calendar have been measured by astronomic observations, however a change occurred in both individual and social focus; from a basis of movements occurring in nature to those of artificial movements, or in other words, from natural cycles to the clock [24,29,39]. Time is thus abstracted as a result of the scientific modalities to measure reality [40]. Therefore, Gardet’s (1975) perspective of time as knowledge is valid for modern science as well [24].

According to Ken Lertzman (2009), there are many broad parallels between traditional resource management systems (TM) and modern science-based resource management (SM) systems. Therefore, it is possible to adopt the same concept for both systems. From this perspective, management systems can be understood as the processes through which the actions, goals and objectives “are legitimized by social norms, values, and institutions, [as are] the actors involved in carrying them out” ([41]: 342). Consequently, the same author concludes that a management system is “the regulation of human behavior in relation to the environment, rather than direct manipulation of the environment *per se*”. As such, we adopt the Lertzman’s concept and in this article will analyze the regulation of the knowledge that supports human relations with the environment [41]. In this sense, we are on the *management of knowledge* level in line with Lertzman’s approach.

The calendar is an instrument of system management. Since the Classical age, the calendar emerges as a way to tame natural time and to control human economic and social activities [28,29]. Therefore, from the “time as knowledge perspective”, when the calendar is used to control natural resources, it is also used to control knowledge regarding time.

Based on the discussion above, we argue that the adoption of the perspective of “time as knowledge” [24], the concept of traditional ecological knowledge (TEK) [9], and the management of knowledge are adequate approaches to meet the objectives of this article. These concepts and approaches allow for the creation of cognitive categories to describe and analyze the pink shrimp fishing calendar constructed by artisanal fishers (cognitive needs) to adapt (dynamic) to the biophysical processes of their territories (local) in the Patos Lagoon estuary. With “time/calendar” as an underlying analytical category and the incorporation of traditional time in the construction of a resource management scenario in the Patos Lagoon estuary, it is possible to diagnose the (in)compatibilities between scientific resource management (SM) recommended by SK and traditional resource management (TM) grounded in TEK.

## Methods

### The Patos Lagoon estuary

**Figure 1:**
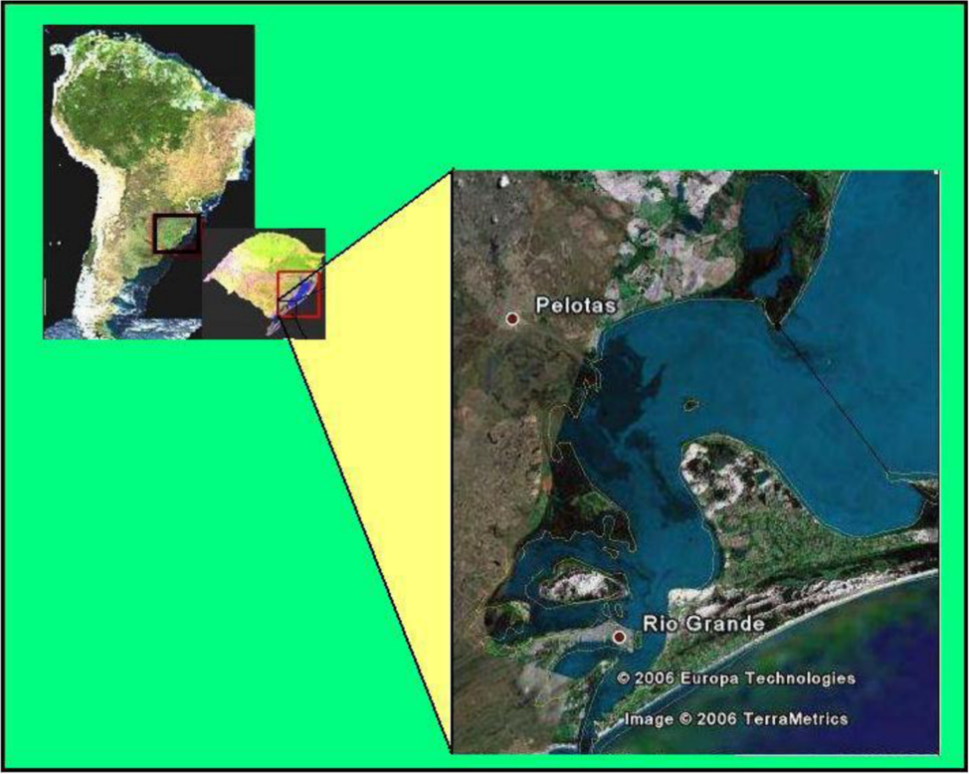
Location of the Patos Lagoon (red square) and the Patos Lagoon estuary (highlighted).

The Patos Lagoon, the world’s largest choked lagoon, is the dominant geographic formation of the coastal plains in southernmost Brazil. It measures 250 km long, 40 km wide, and 5 m deep on average and extends towards the Northeast-Southwest (Latitude 30°30^′^S and Longitude 32°12^′^S). The lagoon receives freshwater from a 201.626 km^2^ drainage basin and in 971 km^2^ of its 10227 km^2^ surface, approximately 10% of its total size, there is mixing of water masses from different origins: freshwater from fluvial origin and sea water from the adjacent ocean [42,43]. These characteristics make this semi-closed water body an estuarine zone [44] (Figure 1).

The interior border of the “estuarine mixing zone” (EMZ; or upper course) of the Patos Lagoon is located at approximately 70 km from the estuary entry (imaginary line which connects the “Lençóis” tip to the “Feitorias” tip (Figure 1). However, this border varies according to high fluvial discharges, which are common at the end of winter and beginning of spring, and to the low to moderate discharges that occur during the rest of the year to the south and north respectively [44,45]. Occasionally, EMZ might infiltrate 150 km into the estuary and might stretch to coastal waters [46]. In El Niño years, there is an increase in fluvial discharges and, consequently, a large plume forms in the continental platform due to the water carried across the Patos Lagoon [47].

Concerning its geomorphology, the Patos Lagoon estuary encompasses two environments: the shallow coastal bays (the protected bay or coves with 1,5 m deep), and the central, deep, open water body (5 m deep), where the central navigation channel is located [45].

A large fish biomass is associated with the high primary productivity in the estuary. The Patos Lagoon is the most important area for the rearing, reproduction, and feeding of a large portion of the existing fish of the southern coast of Brazil [48,49]. In the estuarine region itself, 110 fish species are found, the post-larval, juvenile and sub-adult phases being the most frequently collected [48].

Of these species, only three are commercially exploited by artisanal fishery: the species (*Mugil platanus* and *Micropogonias furnieri*) which spawn in the sea and use the estuarine environment as a larvae and juvenile breeding site (estuary-dependent species) and *Netuna barba*, which spends most of its life cycle in the sea and enters the estuary toward the limnic or pre-limnic zone (anadromous species) [49].

On the other hand, the most economically important fishing resource for artisanal fishery in the Patos Lagoon estuary is the pink shrimp (*Farfantepenaeus paulensis*) [50]. After penetrating the estuarine zone, the shrimp post-larvae grow in the shallow coves, where juveniles enjoy a wider variety of habitats and food resources [51].

The four species cited above have life cycles associated with the hydrodynamic and meteorological variations of the estuary; additionally, their biomasses fluctuate seasonally [49,51]. In the case of pink shrimp, as will be described below, seasonal and inter-annual fluctuations caused by climatic and hydrodynamic variables are factors that influence artisanal fishers’ fishing calendars in the Patos Lagoon estuary.

### History of Fishery Management in the Patos Lagoon estuary

**Figure 2:**
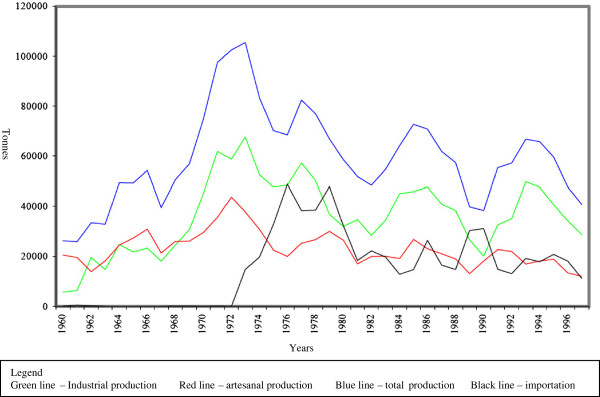
Artisanal, industrial and total production and importation levels of fish in tonnes from 1960 to 1997, State of Rio Grande do Sul (Brazil).

In 1967, the federal government, through the Superintendence for the Development of Fishing (Sudepe), passed the decree, law n° 221/1967, which established the concession of fiscal incentives to large companies in an attempt to make fishing a primary national industry [52]. In the southern region of Brazil, Rio Grande do Sul (RS) stood out as the state receiving the greatest number of incentives to increase fish capture and production [53], especially in the Patos Lagoon estuary and adjacent ocean [54]. As a consequence, the incorporation of modern tools to artisanal fishing practices (fishnets, motors, etc.), the development of new industries, the modernization of existing industries, and the formation of private sea fishing fleets occurred [53,54]. After an increase in production, which for artisanal fishers reached its peak in 1972, and for industrial fishing in 1973, [53], there was a gradual decrease in capture. This led to the bankruptcy of several fishing companies and to the use of increasingly larger fish nets with smaller mesh sizes in an attempt to maintain earlier capture levels [54] (Figure 2).

As a response to collapse of fish stocks, a centralized fishing resources management strategy was developed. Until the mid 1960s standards to regulate fishing in the Patos Lagoon were almost non-existent. After this time, laws meant to impose a specific fishing calendar and fishing techniques, such as those in practice since the late 1960’s, [55] were put into action.

Due to the inefficiency of the centralized resource management strategy, in 1996 a co-management plan composed by different stakeholders (government, local institutions, the fishing community of the Patos Lagoon was created: *The Patos Lagoon Forum* - FLP) [33]. Significant advancements, such as the closed fishing season and the ban on industrial fishing within three miles from the entry of the estuary, have been made following the FLP. At the same time, however, the FLP has achieved poor results concerning discussions on the fishing calendar and permitted fishing techniques.

After the decline of other resources, pink shrimp is presently the main fish resource explored by artisanal fishers. Although shrimp overharvesting is a controversial matter, scientific knowledge produced by researchers from the local university advises a fixed fishing calendar and specific fishing techniques to solve this supposed problem. Despite the formation of the Patos Lagoon Forum (i.e. a co-management scenario), the INC 2004 determines the present use criteria for artisanal fishing in the Patos Lagoon estuary and establishes both a fixed fishing calendar and the fishing techniques, all following the logic of scientific recommendations for the use of this resource [56-59].

The above situation occurs because researchers understand that one of the goals of the Forum is “to provide the fishing community with information about the environment and biology, in addition to providing legal support for cooperative work;” furthermore, it is understood that civil society’s decisions must be “…supported by scientific knowledge and by government agencies” ([60]: 593). According to the wide scientific literature existing in this field [2,12,61], however, this premise distorts the basic principle of dialog between traditional ecological knowledge and scientific knowledge in decision making regarding the management of natural resources in a co-management regime. It is important to stress that the scientific recommendations and those of the INC 2004 disagree regarding the start date of shrimp fishing, despite the consonance concerning the fixed fishing calendar and fishing equipment.

On the other hand, in the second half of the 1990’s see [62-64] and in the first half of the 21^st^ century see [33,54,56], researchers from Rio Grande do Sul who adopt socio-ecological perspectives and a theoretical base to collect ethnoscientific data come onto the scene. Subsequently, they claim that artisanal fishers and fisher-farmers in the Patos Lagoon estuary have a profound knowledge of their natural environment; and that this knowledge must be taken seriously and incorporated into resource management plans implemented by government agencies. This new perspective represents a shift from the view that emphasizes the natural system alone—one often adopted by researchers from the natural sciences who study the fishing resources. Here, these scientists stress the interface between social and ecological systems. From this point on, research on fishing in the Patos lagoon estuary begins to focus not only on natural resources, but also on the fishers’ relation to the local estuarine environment. The co-existence of two opposite theoretical perspectives inside the academy, and within the same resource management scenario, generates discord in relation to the introduction of TEK in public policies regarding natural resource management [65].

The INC 2004 only permits fixed-net and stownet fishing techniques and has additionally restricted fishing to registered “andainas” (bamboo stakes fixed in the lagoon at a depth of up to two meters to which nets are tied); however, several other fishing techniques are still being used by fishers (‘coca’, ‘berimbau’, ‘plancha’, ‘pauzinho’, etc.) in addition to capture in unregistered “andainas” [66]. The same pattern is observed for the fishing calendar: despite the imposition of a fishing calendar by INC 2004, fishers systematically fish outside the legal season [58,66].

From this historical context of dissonance between the fishing calendar based on SK and introduced into law (INC 2004) and the calendar proposed by TEK, both within the from the realm of the Patos Lagoon Forum see [33,56] and from observations made during field research see [58,66], the objective of this article emerges: namely, to discuss the incorporation of traditional time, supported by TEK, in the development of a local resource management scenario. Having reached this goal, the next step is to understand one of the key issues in resource management in the Patos Lagoon estuary; that is, the basis for the dissonance between the fishing calendar prescribed by SK and the one proposed by TEK. After the analysis of this dissonance, we suggest some guidelines for developing this scenario.

### Data collection and analysis

A wide literature review of main publications from the ethnosciences and natural sciences was conducted with the aim of establishing an emic/etic approach to the description of the pink shrimp life cycle and management practices in the Patos Lagoon estuary. The meaning of the word “emic”, which comes from *phonemic* (entonation), describes the behavioral system of a given culture within its own terms. In this case, the cognitive and linguistic categories of a traditional community are being studied. On the other hand, “etic” comes from *phonetic* (written language), which refers to the description of categories of scientific knowledge of nature [67]. In this sense, this article aimed to integrate traditional knowledge into scientific descriptions of the pink shrimp life cycle in the Patos Lagoon estuary.

From the ethnoscientific literature that describes the TEK of the pink shrimp life cycle in the estuary, three articles have been selected [33,56,58], along with a book [66] and a recent Ph. D. (in process of publication) [68]. For the description of TM supported by TEK, the same works are used, as well as a master’s thesis [54]. From the scientific literature, eleven articles were selected, five of which have been published in non-Brazilian journals [69-73] and seven of which were published in Brazilian journals [74-80], as well as two technical reports [81,82], a conference summary [83], two master’s theses [84,85], a Ph. D. thesis [86] and two book chapters [46,51]. For the description of the SM, two additional Brazilian articles upon which the guidelines imposed by INC 2004 were established have been selected [87,88]. It is worth highlighting that: 1 - both the ethnoscientific and the scientific literature selected describe both the pink shrimp life cycle as well as some aspects of the Patos Lagoon estuary’s hydrodynamics; these are necessary to understand the variations in the pink shrimp cycle in the Patos Lagoon estuary; 2 - the time/calendar category is a central analytical category for the discussion on the pink shrimp management.

From the dialog between TEK and SK in the description of the pink shrimp life cycle in the Patos lagoon estuary, the hypothesis of incommensurability at the knowledge level will be tested. From the recommendations stemming from SK and TEK for the use of the natural resource (pink shrimp), the hypothesis of incommensurability at the resource management level will be tested. In this way, the hypotheses tests will “operationalize” the definition of the level(s) at which “qualitatively incompatible principles exist ([89]: 58); in other words, the principles that “generate incompatible descriptions of the reality of nature”. They will additionally show at which levels there are underlying principles of TEK and SK that present similar observations of descriptions of the biophysical environment (if they exist at all)^c^. From “similar observation principles” of the incommensurable level(s), the “underlying principles of TEK and SK” ([14]: 06) emerge, which are considered “criteria for hierarchies of validity” ([34]: 98). According to Santos *et al.* (2005: 53) the “criteria for hierarchies of validity” are used to “compare” types of knowledge “because of their capacity to carry out certain tasks in social contexts delineated by particular logics,” and consequently establish different weights for specific types of knowledge. According to the same author, when there is an “epistemological problem”, relativism, in the absence of “criteria for hierarchies of validity” is an untenable practice because “no human practice could be accomplished” ([34]: 98). In the case of this article, “criteria for hierarchies of validity” will be established with the aim of constructing a “resource management scenario”; specifically they will delimit the discussion of the possibility of the development of a “resource management scenario” for pink shrimp based on the logic of TEK and SK.

## Results

### Describing traditional knowledge and scientific knowledge: the first step in the management of knowledges

The fishers in the Patos Lagoon estuary intensely observe the new moon at the beginning of spring: “The new moon of September is in motion until December”. The first new moon in spring allows us to predict what spring is going to be like in a given year: if it is rainy during the new moon in Rio Grande do Sul, “spring is going to be rainy”. Heavy spring rains in RS result in large fluvial discharges during the entire season in the Patos Lagoon estuary, thus inaugurating what the fishers call a “freshwater year”. If it does not rain during the new moon, spring “is going to be dry”, resulting in low fluvial discharges in the estuary. The low fluvial discharges during spring give rise to two “year categories” depending on the preceding winter. First, if the winter was also dry, a “salty water year” starts; second, if the winter was rainy, the fluvial discharges in the Patos Lagoon estuary decrease in October, and the intrusion of “salty water tips” from the ocean occurs through December (at the latest) giving rise to a “mixed water year” [66]. Several scientific publications [69,70,72,86] corroborate the positive correlations between rainfall and fluvial discharges in the RS basin flowing into the Patos Lagoon. Such correlations also described by the fishers. Inter-annual variations in rainfall have also been observed: peaks in winter (June to August) or in spring (September to November) [82] with the possibility of long drought spells [74] and salty water intrusion as far as 150 km inside the Patos Lagoon [46].

According to local fishers, it is at the unstable moment between the end of winter and the beginning of spring, between August and December, that the “casquinhas”^d^ enter the estuary from the ocean in association with “salt water tips.” [33,56,58,66]. Barcelos (1968), Calazans (1978), D’Incao (1978, 1983, 1984) and Moller *et al.* (2001) register the intrusion of larvae (sub stage 6) associated with the intrusion of salty water, predominantly in the period also indicated by fishers [70,78,80,81,83,84]. However, according to fishers [66] and to the scientific literature [70,78,80,81,83,84], the period when larvae intrude the estuary may vary. If it is a “salty water year”, larvae intrusion may occur continuously from the previous shrimp catch along with the estuarine flood currents generated by the Southern quadrant winds (SE, S, SW), although the highest intrusion levels take place in August [66]. D’Incao (1991: 161) claims that shrimp larvae “intrusions may possibly occur, almost all year round” in the Patos Lagoon estuary. These he believes are associated with the salty water and Southern quadrant winds intrusion, an observation that in partial accordance with the fishers’ reports^e^[88].

The intrusion of larvae, starting in August, associated with salt water intrusion and Southern quadrant winds is documented by Calazans (1978) [83]. If it is a “mixed water year”, the larvae intrusion inside the estuary is subject to the existing freshwater drainage and also to the beginning of salt water intrusion as well as the occurrence of Southern quadrant winds that may occur anywhere between October and December [33,56,58,66]. In line with fishers’ reports, most of the scientific literature [70,71,78,81] registers the intrusion of larvae in the Patos Lagoon estuary between the end of September or beginning of October and December, associated with the intrusion of salt water generated by Southern quadrant winds. If, on the other hand, it is a “freshwater year”, shrimp catches are very unlikely because freshwater strength (high fluvial discharges in the estuary due to the rain) prevent “salt water tips” with larvae from intruding into the estuary before the end of December, a time which is considered the threshold for the occurrence of a good shrimp “catch” [33,56,58,66]. Castello and Möller (1978) and Moller *et al.* (2001; 2009), using statistical analyses from rainfall and catch per unit of effort, have also noticed a negative correlation between high rainfall in the spring, the non-occurrence of Southern quadrant winds, and the abundance of shrimp [70,71,77]. According to Garcia *et al.* (2004), the highest rainfall averages during spring take place in years when there are events such as a moderate to strong El Niño [73]. Therefore, the “freshwater year” from TEK is the (moderate to strong) El Niño year described by SK.

In catch “years”, that is, in “salty water years” and in “mixed water years”, the larvae grow and reach a minimum size which the fishers consider “good”. The appearance of pink shrimp of a “good” size (around 9 mm) depends on the moment when the intrusion of larvae from the ocean has occurred. The shrimp that intrude first will subsequently reach a size “good” size earlier than those that intruded later on [66]. Research aimed at tracing Shrimp biometric curves in the Patos Lagoon estuary have also highlighted the presence of several cohorts (size variation) due to the intrusion of marine waters in the estuary [80].

On the other hand, according to the fishers, pink shrimp does not reach a size that is considered “good” at the same time throughout the entire estuary and, therefore, the fishers establish a zoning (ethno-zoning) in the estuary as follows: The shallow coves are considered “breeding sites”, the main estuarine body is the “passing place” and “the Lagoon” is considered the region above Ilha Nova^f^. In the “breeding sites”, the shrimp reach a “good” size earlier than in any other ethnozone [66]. The “breeding sites” can be broken down into various sectors according to their proximity to the ocean: the fishers believe shrimp tends to reach a “good” size earlier in the embayment areas that are closer to the ocean [85]. Within the scientific literature, D’Incao (1991) and Benvenuti (1998) have observed a higher concentration of juveniles shrimp and pre-adults in shallow coves, in accordance with what is observed by local fishers [51,88]. Although the coves are considered shrimp “breeding sites”, it is worth mentioning that fishers report that the shoals of the banks of the main estuarine are also shrimp rearing areas [68]. D’Incao (1991) also points out the wide distribution of shrimp larvae, juveniles and pre-adults throughout the entire estuary [88].

According to the fishers, once the shrimp reaches a size considered “good”, “the shrimp travels up”, that is, it migrates from the shallow coves (breeding site) and from the shoals across the main estuarine body (‘passing place’) to reach the “mixed water” (similar to EMZ) at larger and deeper sites as it grows in size. On the other hand, this migratory movement down the upper estuary varies between the “mixed water year” and the “salty water year”: in the first one, the “mixed water” and the shrimp remain longer in the “passing places” and in the “breeding sites” next to the lower estuary; and in the second, the “mixed water” is in the “lagoon” and the shrimp “go to the lagoon” as soon as reaches a “good” size [66,68]. Thus, in the “salty water year”, shrimp biomass accumulation occurs in the northernmost parts of the estuarine zone (in the ‘lagoon’) and in the “mixed water year” it occurs in the lower estuary, but in both cases pink shrimp are concentrated in the “mixed water”. In scientific literature, there are no descriptions of pink shrimp migratory movements similar to the ones described by the fishers^g^. However, there is some information in scientific literature that intersects with fishers’ observations. D’Incao (1982) registers high pink shrimp abundance in the Patos Lagoon estuary in areas with salinity levels lower than 10% and higher than 3%. Benvenuti (1978ab) reports pink shrimp movement from the shallow coves to deeper regions due drops in temperature in the fall, thus altering the composition of the benthic communities of shallow waters [74,75,79].

According to local fishers, shrimp returns to the adjacent ocean from March to June, with noted variations in migratory movements between “mixed water” years and “salty water years”. In “salty water years”, migration to the ocean occurs from the “lagoon” passing through the main estuarine body (‘passing places’) during the reproductive migration period. Shrimp reproductive migration occurs during the Southern quadrant calm winds in particular during the waning moon of April and May. Shrimp reproductive migration can take place earlier if rains occur before the waning moons of April and May [66]. In the “mixed water year”, however, the migration does not happen and the migration to the ocean does occur from the “breeding sites” and “passing places” of the lower estuary in a softer and more continuous migratory flow [68]. D’Incao (1991) in his work on the biology of the Patos Lagoon shrimp, describes a shrimp abundance curve in which levels are greatest during the months of March and April and then decrease in May and June [88]. Nevertheless, scientific knowledge is silent regarding migratory movements,’ inter-annual variability, shrimp abundance variation due to heavy rainfall during the end of summer and into the fall, and further fails to consider the relationship between the waning moon and pink shrimp reproductive migration^h^.

### Traditional and the scientific descriptions of management: the second step in the management of knowledges

**Figure 3:**
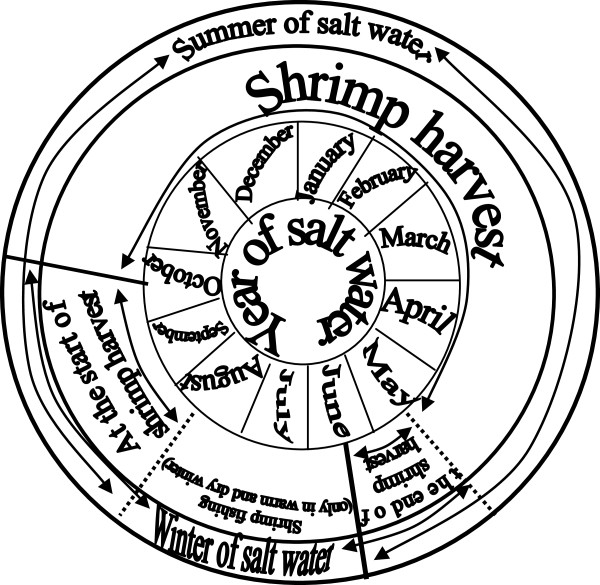
Shrimp fishery calendar in “salty water” years in the Saco do Arraial (shallow water).

**Figure 4:**
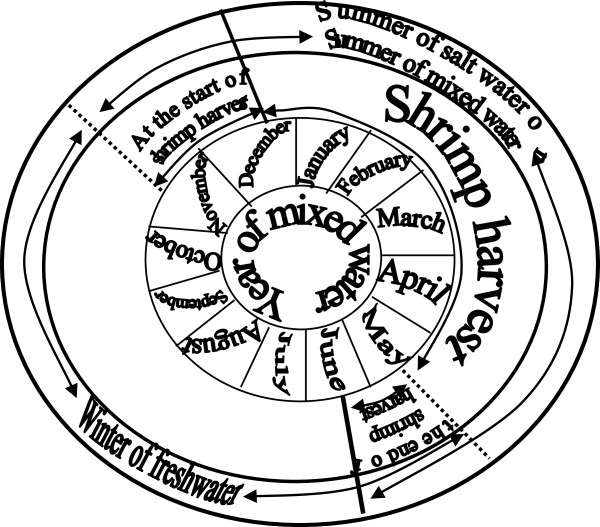
Shrimp fishery calendar in “mixed water” years in the Saco do Arraial (shallow water).

During the initial phases of the pink shrimp catch, fishers are accustomed to “good” sized shrimp [66]. Nevertheless, as was discussed in the previous section, variability in climate and hydrodynamic conditions in the estuary influence the shrimp life cycle in any given year—in addition to the intrusion of larvae in the “salty water tips” and, consequently, the moment at which shrimp reach a size that is considered “good”. In “salty water years”, the shrimp can reach a “good” size as early as September and, as a result, shrimp catch activities initiate sooner [66]. There is still another possibility: in “salty waters years”, if the winter is warm and dry, larvae that have continued intruding the estuary since their previous reproductive migration period can reach a “good” size in the winter. This thereby allows virtually continuous shrimp fishing activities in the estuary [33,66] (Figure 3). However, in “mixed water years”, the onset of fishing activities depends on “good” shrimp emergence, which occurs from November onward, but can also occur in January (Figure 4) [66]. Therefore, the fishers’ calendar varies according to the known seasonal and inter-annual estuarine-biological dynamics, which allows them some degree of predictability.

Moreover, the beginning of pink shrimp fishing activities in the Patos Lagoon estuary is not the same for all fishing communities. The beginning of fishing activities described above is based on the research by Moura (2009) in a fishing community whose traditional territory is located on a shallow cove (breeding site) of the lower estuary. Here, shrimp reaches a “good” size earlier than in other ethnozones [66]. According to other ethnoscientific studies [56,58,68] carried out in fishing communities with traditional territories in the main estuarine body (‘passing places’), the beginning of the shrimp catch may occur from the end of November or December in “salty water years” or in January or February in the “mixed water years”. In this sense, the fishers from these “passing places” would catch both good-sized shrimp “traveling” from “breeding sites” towards “mixed water zones”, which have “reared” on the shoals at the banks of the main estuarine body, as well as shrimp in the ocean entry [68]. In fishing communities next to the northern border of the estuary (‘the Lagoon’s mouth’), such as São Lourenço do Sul, the “salty water years” provide climate and hydrodynamic conditions that make them “passing places” for shrimp. As a result, fishing calendars similar to those developed by the fishing communities from “passing places” of the lower estuary are noted. However, in “mixed water years”, the estuarine dynamics differ from the dynamics of the lower estuary communities [54]. Therefore, more detailed studies are needed to understand the interactions between shrimp catch productive systems, traditional calendars, and estuarine dynamics.

The same flexibility that marks the beginning of fishing activities is also true for the factors that determine the closure of the pink shrimp catch season. In “salty water years”, the end of the catch occurs when “lagoon” shrimp (shrimp from upper estuary) begin their reproductive migration to the ocean (passing by the main estuarine body, ‘passing places’, during the waning moons of April and May). In any of the “years”, however, the end of the season can be earlier if it rains in excess [66]. Although the calendar incorporates environmental variability, the end of the catch occurs at the same time in virtually all the fishing communities. This can be observed by the fishing calendars of communities with traditional territories in regions of “breeding sites” see [66], and in the main estuarine body (‘passing places’) see [56,58]. It is worth highlighting, however, that fishing communities located close to the northern border of the estuary (‘the lagoon’s mouth’), like in São Lourenço do Sul, salty water intrusion occurs less frequently than in lower estuary communities [54]. Therefore, research must be conducted to better understand these groups’ fishing calendars in “mixed water” years.

The scientific literature recommends that shrimp harvesting begin at a fixed date, the beginning of March, across the entire Patos Lagoon estuary. The literature cites two motives for this recommendation: 1 - to avoid high capture rates of shrimp reproductive matrixes that migrate to the ocean, and 2 - to allow fishing in the months when shrimp is in higher abundance. Abundance numbers are based on the statistical averages of annual series studied in the estuary [87,88]. D’Incao (1990; 1991) recommends the closure of shrimp capture between the end of May and the beginning of June for the entire estuary, based on statistical averages that show a sharp decline in pink shrimp abundance during this period [87,88].

## Discussion

### Traditional knowledge and scientific knowledge: one or two descriptions of the pink shrimp migration cycle in the Patos Lagoon estuary? The third step in the management of knowledges

The dialog between TEK and SK allows us to observe similarities and differences in the two “realities” described for the pink shrimp migration cycle. One observed difference is that the first is holistic; it makes connections between several elements of nature (new moon in September, rains and pink shrimp migratory cycles; April and May waning moons, Southern quadrant winds and the shrimp migratory cycle). These connections are overlooked by the positivist SK. SK’s analytical-reductionist base and TEK’s holistic base have also been noted by authors, such as Berkes (1999) and Vieira *et al.* (2005) [1,9]. Furthermore, TEK reveals a migration pattern (from lower estuary to upper estuary) and inter-annual variability in pink shrimp migration movement (in “freshwater year”, “mixed water year” and “salty water year”) [66], which were not predicted in the scientific literature. Johannes *et al.* (2000) has also observed migration patterns and time variability in whale and fish stocks noted by TEK, but not predicted by SK [16].

Despite these differences, significant consonances are observed and show a likeness between the “realities” described by SK and TEK. These agreements allows us to trace two common and fundamental principles for the construction of “resource management scenarios”: the spatial heterogeneity (shallow coves, EMZ, and main estuarine body) and the environmental variability and flexibility (inter-annual variations, rainfall and winds regime in spring, existence of several shrimp cohorts, etc.). These two principles will be the basis from which we discuss TEK-based management and SK-based management in the next section of this argument.

### Discussions of traditional management practices and scientific management recommendations: the fourth step in the management of knowledges

Based on the previous discussions, we understand that fishers of the Patos Lagoon estuary construct calendars/time based on material references from nature (pink shrimp appearance and disappearance), which are linked to the framework of material references of estuarine-biological processes (‘salty water tips’, waning moons, winds, etc.), which according to TEK favor shrimp occurrence. In this sense, in TEK there is a time/space overlap. According to Chesneaux (1989) and Giddens (1991), time located in its natural environment is flexible and irregular, and allows the ecosystem to “take its time” [26,28]. In the case of fishing communities in the Patos Lagoon estuary, besides the space/time intersection, which provides flexibility within the traditional calendar, ecosystem time is anchored upon the idea of “good” shrimp size. To this end, fishing communities’ social value of the “good” shrimp size guides the relationship between man/shrimp/estuary. According to Stevenson (1996), rules and values driving a relationship considered socially appropriate between man/environment is called an “ethic code” [90]. In this case study, fishers’ “good” shrimp size is an “ethical code” that establishes a minimum size level for the catch. It is a measure of what is socially acceptable concerning the use of this resource. The “ethic code” engenders the necessary amount of time for estuarine-biological dynamics to occur, favoring the development and the appearance of a socially valued minimum-sized shrimp.

The spatial heterogeneity observed can also be understood from the perspective of time in the natural environment. Trusler and Johnson (2008) argue that traditional human groups appropriate the heterogeneity of terrestrial landscapes because they manage resources that are dependent on potential seasonal catches [91]. Acheson and Wilson (1996) demonstrate that environmental parameters often guide management of fishing resources by artisanal fishers’ communities [92]. In the Patos Lagoon estuary, “the ethic code” is followed via the management of hydrodynamic, biological, and climate conditions, which allow the “good” shrimp acquisition in several fishing territories located in their respective ethnozones.

Based on variations in estuarine-biological dynamics, the fishers in the Patos Lagoon estuary construct their time/calendar for their respective fishing territories. According to Allut (2000), TEK allows the fisher to organize his world, and from this order, construct scenarios for actions that are both organized and adaptative to his surroundings and constantly changing [38]. Steward (1955:31) proposes “ecological scenarios” in which man inserts himself with his “superorganic factor” [93]. In this sense, it can be argued that “salty water years”, “freshwater years” and “mixed water years” are not just cognitively constructed calendars, but “ecological scenarios” constructed by fishers in the Patos Lagoon estuary based on their TEK, allowing for their insertion and adaptation into the estuarine-biological dynamics of their fishing territories.

Although SK and TEK display common ground regarding the shrimp migration cycle, SM guidelines present an entirely different management scenario from the management practices carried out by traditional fishing communities in the Patos Lagoon estuary. Researchers recommend pink shrimp capture permissions/prohibition across the entire estuary during the same time periods with fixed start and end capture dates (see D′Incao, 1990; 1991). These recommendations are based on statistical averages that do not differentiate between different ecological domains; they also fail to consider the seasonal and inter-annual variations of shrimp life cycles in different domains (shallow coves, EMZ and main estuarine body), something that is accepted by INC 2004.

In the case of SM and OM in the Patos Lagoon estuary, the technical and scientific modality of mediating reality through techno-social time abstracts estuarine-biological dynamics, which are described by scientific knowledge itself. The fixed time measurement, alien to natural rhythms, makes time empty [28] and is a consequence of the technical model of mediating reality [40]. According to Chesneaux (1989), within fixed/rigid time (‘techno-social time’), the flexible functioning of biological rhythms is increasingly overlooked, especially for human actions. It is also imposed by INC 2004 and supported by scientific recommendations for fishers in the Patos Lagoon estuary.

Another contradiction refers to the different ecological domains noted and the possible time variations we described in the previous section. According to its recommendations, SM standardizes the space of the Patos Lagoon estuarine and applies a fixed time management regime to all ecological domains. For Trusler and Johnson (2008), the standardization of biological and physical properties of a certain ecological domain pertains to Cartesian reasoning, which understands space as an abstract entity [91]. In this sense, the emptying/abstraction of time is a pre-condition for the emptying/abstraction of space in the SM propositions in consonance with Giddens’ (1991) discussion on modern time-space [28].

Therefore, we can say that at the level of resource management, there is incommensurability based on SK and TEK due to the incompatible concepts between the two forms of resource management. Traditional resource management obeys “ecological scenarios” constructed according to estuarine-biological dynamics in several ethnozones acknowledged by TEK. On the other hand, official resource management follows the recommendations of scientific studies which abstract the seasonal and inter-annual variability across distinct ecological domains as predicted by SK; this occurs through the use of statistical averages of pink shrimp abundance curves. Trusler and Johnson (2008) similarly show dissonances between the Gitksan Wet’suwetèn natives’ management and that recommended by the BC Forest service of Canada due to differences in concepts regarding the biophysical environment. For the former, the biophysical environment is appropriated and managed according to the “place” concept (known and experienced ecotopes, immersed in the group’s social relations). However, for the latter, the environment is appropriated and managed according to the Cartesian reasoning of abstract space [91].

### (De)constructing a management scenario for pink shrimp: fifth step for the management of knowledges

The federal government chooses to adopt SK recommendations for pink shrimp management in the Patos Lagoon estuary. According to Berkes and Folke (1998), centralized management in institutional bureaucracies is characteristic of resource management based on Newtonian sciences [2]. By choosing this authoritarian management style, Ibama’s institutional bureaucracy applies what Elias (1998) has described as ‘space-time control at an abstract level’ [94]. Both the diversity in ecological domains and the environmental dynamics in the Patos Lagoon estuary are abstracted by the recommendations of SM. Moreover, SM is at odds with what is imposed by daily TM actions based on TEK carried out by fishing communities in the Patos Lagoon estuary. This “misalignment” created by the space-time abstraction of the quotidian actions is what Chesneaux (1989) calls a “disruption between man and space-time” [26].

Due to incommensurability at the management level, conflicts arise between fishers from several communities of the estuary and institutional bureaucracies see [58]. To resolve this impasse, with the aim of constructing “resource management scenarios”, we adopt Kuhn and Feyerabend’s perspective, stressed by Oberheim (2004): we only have the ability to perceive certain meanings in the world after we are in possession of a particular theory [95]. In the case of this article, estuarine-biological pattern perceptions occur through the sameness of the “realities” described by SK and TEK, which allows us to grasp two common and fundamental principles for the construction of “resource management scenarios”: spatial heterogeneity and environmental variability and flexibility. According to Santos *et al.* (2005), it is necessary to establish “criteria for hierarchies of validity” when an “epistemological problem” exists and interferes in human activity [34]. In this sense, the two principles represented in both SK and TEK are taken as “criteria for hierarchies of validity” in the construction of resource management scenarios for pink shrimp in the Patos Lagoon estuary. This is simply because there is incommensurability at the resource management level between the scientific and traditional knowledges. Thus, in the construction of a possible management scenario for pink shrimp in the Patos Lagoon estuary, these two principles underlying both SK and TEK must be considered (‘criteria for hierarchies of validity’) which emerge from “similar observation principles”. As shown in the previous section, as opposed to OM which follows scientific recommendations, TM complies with these two common principles and is thus more similar to “reality” (estuarine-biological patterns) because it complies with the predicted estuarine-biological dynamics according to TEK and SK. Accordingly, Berkes and Folke (1998) claim that TM incorporates active adaptations to the known natural environment [2].

Based on the presented arguments, it is possible to raise some hypotheses based on TEK for the investigation by SK as a first guideline for the construction of pink shrimp management scenarios. Thus, we see roles changing places, as TEK complements SK in several scientific bibliographies see [2,10]. In this case study, SK is understood as complementary to TEK in the construction of management scenarios for the pink shrimp in the Patos Lagoon estuary. When these hypotheses are investigated by SK, they can contribute to SK advancement in two ways:

a) To enrich scientific descriptions of the pink shrimp life cycle in the Patos Lagoon estuary by adding the following information: top/down estuary migration; biomass concentration in the “mixed water”/brackish water; the life cycle phase that involves the migration from the lower estuary embayment areas to the upper estuary; inter-annual variations in the migration cycle and in biomass distribution, especially when EMZ is closer to the embayment areas (‘mixed water year’) of the lower estuary or when it is farther than 70 km inside the estuary (‘salty water year’); variations in spatial distribution and abundance due to rainfall during summer and/or fall; correlation between long drought spells and pink shrimp presence in the estuary once the indispensable conditions for larvae intrusion predicted by SK and TEK are met; correlation between the new moon and rainfall levels in the spring; correlation between the waning moon, winds, and pink shrimp reproductive migration.

b) To change the scientific paradigm from positivism to holism: besides the correlation of a number of environmental factors, as cited in the previous section, other factors contribute to the shift from a more positivist science to a more holistic science; for instance, to broaden the focus to include the socio-ecological system, which allows for natural environment; to further delimit gaps in SK by means of TEK and acknowledge the existence of an ethical code in the TM. Therefore, a focus on the socio-ecological system is proved most adequate for natural resources management as compared to scenario that only includes the natural system.

The cited ways in which TEK contributes to SK are also registered in the ethnoscientific literature [7,9,13,14,16,18,19]. According to Huntington (2000), Johannes *et al.* (2000) and Ruddle (2000), TEK provides valuable information about stages in marine resources migration cycle, which may contribute to SK in the mapping of marine resources distribution and migration cycle in resource management scenarios [13,16,18]. In the case of pink shrimp in the Patos Lagoon estuary, the investigation of information coming from TEK by SK is important for the construction of “management scenarios” since they provide action guidelines in TM. TEK, like SK, is subject to errors [13,16] and the investigation of such situations through SK in “management scenarios” forges trust in both kinds of knowledge and opens up new uncertainties and hypotheses. Moreover, an attempt to change scientific paradigm through the correlation of several environmental events is, according to Huntington (2000), a way to change the amoral and positivist occidental paradigm regarding man/natural environment relationship to a more ethical and holistic relation [13]. In “resource management scenarios”, a holistic view and a more ethical relation with nature are widely recommended in the scientific literature see [9,14].

Besides the above recommendations for the natural sciences, we also have some recommendations for the interdisciplinary and social sciences. According to our literature review, we can observe some gaps in discussions of the traditional calendars of different fishing communities in the Patos Lagoon estuary. The available information comes predominantly from the lower estuary communities whose territories are limited to borders of the estuary. Thus, research on traditional calendars are needed in fishing communities whose territories encompass embayment areas, the main estuarine body of the upper estuary, and the flexible borders of the estuarine zone, such as communities at the northernmost part of the Patos Lagoon. These communities are often located near the EMZ. Moreover, little information exists for the southernmost communities, which are closer to break waters and where residents also fish in the ocean.

Once the above guidelines imposing limits to SK in the initial construction of “management scenarios” are taken into account, it is possible to trace two additional guidelines derived from the discussions thus far and from the two “criteria for hierarchies of validity” that have arisen from the interaction between SK and TEK. The first arises from the spatial heterogeneity existing in the Patos Lagoon estuary. The Patos Lagoon estuary should be seen and managed as an ecological mosaic. In the estuarine zone there are several ecological domains (shallow bays, EMZ, main estuarine body, lower estuary, upper estuary) which contribute to pink shrimp development during different life cycle phases. Thus, it is an error to adopt a closed fishing season for the entire estuarine body as SM prescribes. For the different ecological domains described, where the pink shrimp reaches a size considered “good” at different moments, there should be different permitted fishing seasons. The lower estuary embayment areas, for instance, are apparently the first ecological domains where pink shrimp reach a “good” size. Therefore, this community could be given permission to catch pink shrimp earlier than the other communities. In Mamirauá Sustainable Development Reserve, state of Amazonas, considered one of the icons in the participatory and adaptative resource management in Brazil, natural resources management obeys an environmental mosaic constructed from the heterogeneity of the ecological domains known by SK and TEK [96,97].

The second guideline arises from the seasonal and inter-annual variability of estuarine-biological force mechanisms. According to SK and TEK, there are several environmental force mechanisms that influence the conditions allowing for the appearance of a “good” sized shrimp (or 9 mm) in variable inter-annual periods. Therefore, it is a mistake to adopt a fixed fishing calendar, according to what SM purposes. The permission or prohibition to catch shrimp should be flexible, being determined annually according to the appearance or disappearance of pink shrimp of a “good” size. In this case, the extension of the closed fishing season, and the 4-month closed season, fall short of considering the social and biological-estuarine context. An estuarine-biological context, in which both could be extended, for example, is the moderate to strong El Niño year (‘freshwater year’, for TEK) in which both kinds of knowledge acknowledge the non-occurrence of pink shrimp catches. Wilson *et al.* (1994) and Acheson and Wilson (1996) say that the “parametric management” carried out by fishers through the physical-biological parameter variability responds to the environmental complexity. It keeps the environment inside a chaotic variation spectrum [92,98]. At the Lagoa do Peixe Forum (RS) the beginning and the extension of pink shrimp harvest are flexible dates and are determined at the Fishing Forum^i^ according to that meteorological and hydrodynamic conditions of the current year.

It is worth stressing, however, that the proposition of this “management scenario” for pink shrimp in the Patos Lagoon estuary does not replace the participation of traditional populations in decision making processes regarding management, as is recommended by the co-management bibliography see [2,15,33]. We make this argument because of the legitimate right of traditional people to actively manage their territorial resources [12]—in addition to the de-codification problems in etnoscientific studies see [16], the transformations in the study communities over time (studies are timely), and the fact that studies do not encompass all the social interests of these groups.

## Conclusion

The description of the relational dynamics of hydrodynamic processes and pink shrimp migration cycle in the Patos Lagoon estuary based on TEK and SK is compatible. Despite the compatibility at the knowledge level, TM and SM are incommensurable and point to distinct ways of using the resources of *Farfantepenaeus paulensis*.

The incommensurability at the pink shrimp management level is due to the foundations used to construct traditional and scientific knowledge and management regimes. TEK and the TM are based on the construction of “ecological scenarios”, that is, on a perception of time-space located in the local ecosystem allowing for the adaptative insertion in a known natural instance. SK and the SM are based, on the other hand, on modern time-space perceptions that are guided by Cartesian reasoning that sees time in a homogeneous, dislocated/abstracted way within the Newtonian way of authoritarian resource management centralized in institutional bureaucracies.

Despite the incommensurability at the management level, in the case of the Patos Lagoon estuary, it is possible to establish guidelines for the construction of “management scenarios”. These can be derived from the establishment of “criteria for hierarchies of validity” formulated from common principles that have emerged from the dialog between SK and TEK in the description of the estuarine-biological force mechanisms.

Since TM entirely meets the “criteria for hierarchies of validity”, and consequently the proposed “management scenarios” are in accordance with TM, several hypotheses have been raised for SK, such that new hypotheses, information, and (un)certainties arise.

Despite the “management scenario” proposition, it is recommended that the co-management process adopted by the Patos Lagoon Forum truly incorporate TEK in future public policies for the resource management in the Patos Lagoon estuary. This stands in opposition to the present authoritarian policy centered on institutional bureaucracies and positivist science.

The results of this article should be used legitimate the discussions regarding the management of fishing resources in the estuary held by of the Patos Lagoon Forum. Such valorization is needed to move away from the authoritarian role that SK has played in guiding the official management of the INC 2004, and to construct new “resource management scenarios” with popular participation. The institutions in charge of institutional bureaucracies should be prepared to develop mechanisms to adapt official management plans, according to the timing flexibility and special heterogeneity highlighted by the “criteria for hierarchies of validity”. These emerge and can emerge hereafter from a working dialog between SK and TEK, which today only TM currently meets. Thus, the traditional resource management has a role to play from which it should never be alienated: maintaining autonomous and legitimate guidelines for the management of the territorial resources of artisanal fishers and fisher small-farmers in the Patos lagoon estuary.

## Endnotes

^a^Portaria n. 001, de 02 de janeiro de 1973. Governo Federal: Ministério da Agricultura - Superintendência do Desenvolvimento da Pesca (SUDEPE).

^b^Instrução Normativa Conjunta nº 3 de nove de fevereiro de 2004. Governo Federal: Ministério do Meio Ambiente e Secretaria de Aquicultura e Pesca (Federal Government: Ministry of the Environment and Secretary for Fish and Aquaculture).

^c^Two assumptions derive from the theoretical base and the methodological procedures adopted in this article: 1 - regarding incommensurability at knowledge and management levels, there will be no “similar observation principles” because, according to Feyerabend (1962: 68/94), when theoretical perspectives provide incompatible descriptions of reality, they are mutually exclusive [89]; 2 - regarding incommensurability at the knowledge level, “similar observation principles” can exist. The first assumption is a theoretical consequence and the second one is our postulate.

^d^It is possible that “casquinhas” are equivalent to what science call pink shrimp post-larvae (from sub-stage 6 on), but investigations are necessary in order to confirm this.

^e^It is worth stressing that the hypothesis raised by D’Ìncao (1991) is based on Brisson’s (1977) study, which highlights the intrusion of shrimp post larvae all year round in Laguna de Aruana, Cabo frio (Rio de Janeiro) [88,99].

^f^The border established by the fishers as the beginning of the “lagoon”, the “lagoon’s mouth”, which would be on Ilha Nova, is located very close to the border established by the scientific literature, Ponta dos Lençóis and Ponta da Feitoria, for the end of the estuarine area of the Patos Lagoon. In both cases, the distance between the communication area of the Patos Lagoon and Ponta dos lençóis or the “lagoon’s mouth” would be approximately 70 km (for the scientific delimitation, see [46]). For the fishers’ delimitation, see Moura (2012; *in print*) [66,68].

^g^The oversight of the scientific literature on the issue of down estuary migration probably occurs due to sampling patterns. Almost all the research on pink shrimp in the Patos Lagoon estuary has restricted its sampling to the shallow coves of the lower estuary see [78-80,84,87,88].

^h^After pink shrimp reproductive migration from the estuary to the adjacent ocean, TEK and SK do not present certainties regarding reproduction areas and migratory routes. SK merely mentions Santa Catarina’s coast as a single possibility see [88].

^i^Lagoa do Peixe Forum is a local co-management forum. It can be stressed, however, that despite the existence of this forum and the adaptive character in pink shrimp management, the Park Administration, following attachment 10 D from the management plan see [100], develops a policy of banning artisanal fishing within the park borders (see [27,101,102]. We vehemently disagree with the policy of banning fishers within the park.

## Competing interests

The authors declare that they have no competing interests.

## Authors’ contributions

GGMM has made substantial contributions to conception, design, data acquisition, analysis, and interpretation. He has also been as involved in drafting this manuscript and revising it critically for its intellectual content. Moreover, he worked in the acquisition of funding. DCK has made substantial contributions to conception and design as well has been involved in drafting and revising the manuscript for intellectual content. Moreover, she worked in the general supervision of the research group (Network for Collaborative Coastal Management) and in the acquisition of funding. ACSD has made substantial contributions to the conception, design and interpretation of data and has been involved in drafting and revising the manuscript for its intellectual content. All authors have given approval for the publication of the final version.
